# The Value of Multiple‐Generation Cohorts for Studying Parenting and Child Development

**DOI:** 10.1111/cdep.12403

**Published:** 2021-03-22

**Authors:** Tina Kretschmer

**Affiliations:** ^1^ University of Groningen

**Keywords:** cohort, intergenerational, longitudinal

## Abstract

Participants in longitudinal studies that followed children into adulthood now have children of their own, which has enabled researchers to establish multiple‐generation cohorts. In this article, I illustrate the benefits of multiple‐generation cohort studies for developmental researchers, including: (a) the impact of child and adolescent characteristics (i.e., preconception factors) on parenthood can be studied from a developmental perspective and without having to rely on retrospective reports, (b) intergenerational continuity and transmission can be examined for psychological, behavioral, and social development, and by comparing parent and offspring generations for the same developmental period, and (c) the interplay of genetic and environmental influences on parenting and child development can be disentangled. Even though multiple‐generation studies pose unique logistical and methodological challenges, such cohorts are indispensable for rigorous research into parenting and the origins of child development.

Longitudinal cohorts provide the opportunity to track development over time and offer rich information on associations between childhood exposure and later outcomes, and about stability and change in health and behavior. Such studies have contributed to our knowledge about precursors of health, substance use, and socioeconomic status (Evans‐Lacko et al., 2017; Hobcraft & Kiernan, 2001; Maggs, Patrick, & Feinstein, 2008; Montgomery, Bartley, Cook, & Wadsworth, 1996), and represent an important methodological foundation for developmental research.

Participants in several cohorts from the 1990s and early 2000s have grown up and had offspring of their own, which greatly increases the value of these cohorts for developmental researchers. Data from multiple‐generation cohorts can be used to improve and extend research into (a) preconception influences on parenthood, (b) intergenerational mechanisms in psychological, behavioral, and social development, and (c) the interplay of genetic and environmental influences on parenting and child development. It is puzzling that multiple‐generation cohorts are not used more often to study parenting and child development in a rigorous developmental framework and without having to rely on retrospective data.

With an eye toward changing this, in this article, I introduce examples of current cohorts, then discuss concrete research problems for which multiple‐generation cohort data can allow for improved design and greater methodological rigor, or provide the opportunity for novel insights. My discussion focuses on social development and mental health, but I also provide examples of a broader range of possible research questions, as well as a brief description of how multiple‐generation cohort research can inform practice. In doing so, I hope to convince developmental researchers of the treasure trove of multiple‐generation cohort data and encourage greater use of such data in studies on parenting and child development.

## Multiple‐Generation Cohorts

In addition to focal participants (i.e., the participants who are the focus of a study, or G1), longitudinal cohorts sometimes include parents (G0) or offspring (G2). The G0–G1 design is common in pregnancy/birth cohorts, and can include information collected from parents (G0) as well as information on focal participants (G1). Such a design often allows researchers to collect rich data on the gestational period and G1 development, but does not provide information on G0 experiences that precede conception. For instance, pregnancy/birth cohorts usually do not contain preconception data on exposure to stress of G0s as children and adolescents, which is problematic because early adversity affects individuals across the life course (Entringer et al., 2011; Raposa, Hammen, Brennan, O’Callaghan, & Najman, 2014; Solís et al., 2015). We do not know how preconception experiences, including not just adversity but also symptoms of psychopathology, might be stored psychologically and biologically, and how they might affect parenting and the development of offspring. Pre‐existing risks that do not flare up during pregnancy might easily be missed, even when researchers try to assess developmental histories of parents, because retrospective recall is often biased (Hardt & Rutter, 2004). Finally, pregnancy cohorts usually focus on biological mothers, omitting a comprehensive understanding of the impact of both parents’ health and lifestyles on birth outcomes, parenting, and child development.

To understand how preconception experiences affect parenting and child development, we need multiple‐generation cohorts in which G1 are studied prospectively from childhood through to pregnancy and parenthood—which contain detailed information on G1’s development *long before becoming parents—*and that also include data on G2 that are collected prospectively. Such cohorts allow for rigorously testing mechanisms, as proposed in Belsky’s determinants of parenting model (Belsky, 1984); in this model, developmental histories of parents, along with personality and psychopathology, are highlighted as parental characteristics that influence parenting and ultimately, child development. Compared to personality and psychopathology, developmental histories are neglected in research (Taraban & Shaw, 2018), which underlines the need to use multiple‐generation cohorts.

**Figure 1:**
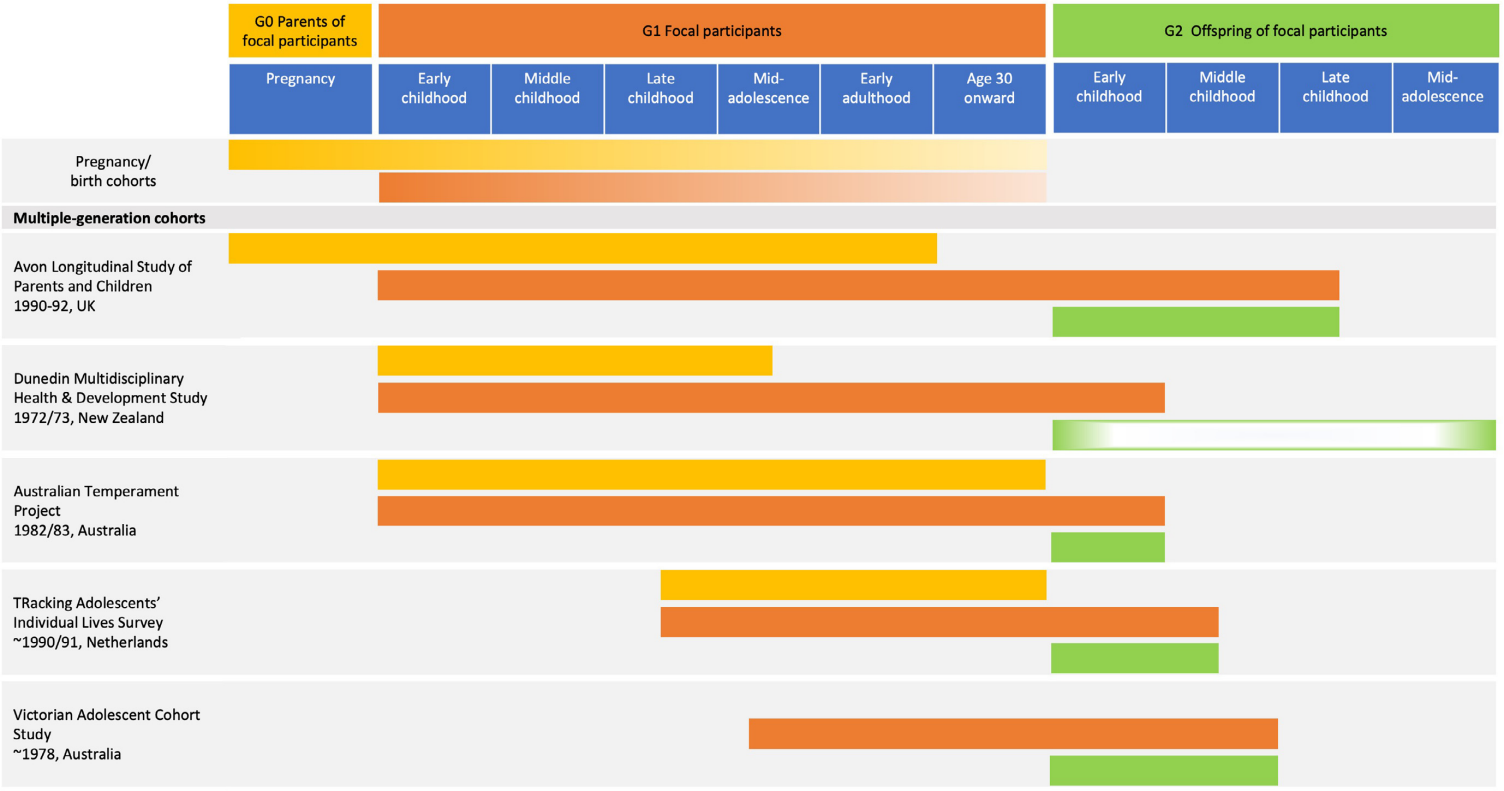
Current multiple‐generation cohorts. This figure provides original cohort names; names of next‐generation cohorts and links to study websites can be found in Supporting Information. [Color figure can be viewed at wileyonlinelibrary.com]

Fortunately, such cohorts exist because ongoing longitudinal studies include offspring (G2), with regular assessments of both G1 parenting and G2 development at several points during G2 childhood and adolescence. Among studies of this type are the Dunedin Multidisciplinary Health and Development Study (Poulton, Moffitt, & Silva, 2015), the Avon Longitudinal Study of Parents and Children (Boyd et al., 2013; Lawlor et al., 2019), the Victorian Adolescent Health Cohort (Spry, Olsson, et al., 2020), the Australian Temperament Project (Olsson et al., 2020), and the TRacking Adolescents’ Individual Lives Survey (Oldehinkel et al., 2015). Figure 1 depicts the developmental periods of G0, G1, and G2 that are covered in these cohorts; see Supporting Information for more information.^1^

These cohorts feature (a) preconception information continuing at least into adolescence, (b) boys and girls to ensure natural recruitment of fathers into the cohorts, and (c) data on parenting and G2 psychological, behavioral, or social development. As such, they overcome common limitations of pregnancy cohorts and are particularly valuable to developmental researchers. However, more studies include offspring of G1, such as the Pittsburgh Girls Study (Hipwell, Tung, Northrup, & Keenan, 2019), the Research on Adolescent Development and Relationships study (Branje & Meeus, 2018), and the Quebec Longitudinal Study of Kindergarten Children (Carbonneau, Vitaro, Brendgen, & Tremblay, 2018). Even twin cohorts such as the Twins Early Development Study and the Netherlands Twin Register collect data on offspring. Next, I discuss how these cohorts help developmental researchers.

## The Impact of Child and Adolescent Characteristics on Parenting

Research on antecedents of parenting, the quality of parent–child relationships, and parents’ well‐being tends to focus primarily on the personalities and mental health of parents or offspring and on environmental conditions, but rarely considers the development and experiences of parents prior to conception (Taraban & Shaw, 2018). However, in studies that have used multiple‐generation cohort data, G1 adolescent mental health is associated with antenatal mental health problems among fathers and more problems among mothers in bonding with offspring (Borschmann et al., 2019; Spry et al., 2018). Thus, parents’ mental health prior to conception is a precursor to the quality of parenting and parents’ mental health, which in turn might affect the development of offspring.

As another example, imagine parents who, as children, were bullied by peers. Not everyone who has been subjected to negative interactions with others displays psychological maladjustment, but peer experiences might affect parenting (Barnett & Taylor, 2009). Parents who were bullied might have derived a hostile attribution bias from their experiences and become overly protective of their offsprings’ peer interactions. Indeed, in one study, mothers’ recollections of their own peer relationships were linked to their childrearing intentions and parenting behaviors (Putallaz, Costanzo, & Smith, 1991), and to their interpretations of their offspring’s peer interactions (Putallaz, Klein, Constanzo, & Hedges, 1994). Specifically, mothers whose recollections of peer relationships were anxious and lonely took a more active role in peer‐related parenting, which suggests that reflecting on one’s ghosts from the past and developing constructive coping mechanisms could provide a target for parenting interventions.

Multiple‐generation cohort data allow researchers to study links between G1 mental health and experiences in social relationships during childhood and adolescence, and parenting later on. Given that multiple‐generation cohorts often contain *repeated* measures of social, behavioral, and psychological constructs, researchers can even explore how stability and change are associated with parenting; this is because the developmental trajectory of an experience or symptom might be a more powerful antecedent than a snapshot taken once during childhood or adolescence, or retrospective assessment.

## Intergenerational Transmission of Normative and Nonnormative Aspects of Development

G1–G2 designs are most informative when G2 assessments are conducted at specific ages of offspring rather than as “by‐catch” during regular parent assessments (i.e., researchers collect G2 data when they collect G1 data because it is convenient to do so). This allows researchers to link G1 development at a specific age to G2 development *at the same age;* it also enables researchers to make inferences about intergenerational transmission of social, behavioral, and psychological concepts. This type of research is needed because contemporary work is often based on retrospective reports. For instance, research on intergenerational transmission of the quality of parent–child relationships often relies on recollections of earlier experiences by caregivers and their association to concurrently measured experiences or outcomes in offspring (Wu, Zhang, & Slesnick, 2020). Similarly, research on intergenerational transmission of mental health often uses parents’ retrospective accounts or a concurrent assessment, but does not compare G1 and G2 at the same age (see Johnston, Schurer, & Shields, 2013, for an exception using G0–G1 data from the 1970 Birth Cohort Study).

However, recollections may be selective and colored by current mental health and cognitions pertaining to one’s own parenting approach. Although not limited by retrospective reporting, comparing assessments of adults’ (i.e., parents’) mental health or relationship quality to those of children (i.e., offspring) is like comparing apples to oranges. Not only do researchers use different instruments to assess adults and children, but the concepts of interest also have distinct meanings at different developmental stages.

Research on intergenerational transmission benefits greatly from the availability of preconception information in the parent generation, not only because this adds rigor to this type of research but also because it can shed light on specific transmission patterns. For instance, adolescent offspring in the Dunedin study were at particular risk for maladjustment when parents had been diagnosed with depression in childhood or adolescence and experienced two or more episodes (Jaffee et al., 2020). Researchers could explore the role of possible “transmission belts,” that is, conditions that are favorable to intergenerational transmission (Schönpflug, 2001). Using the earlier example of negative peer experiences in G1 as a predictor of overprotective parenting of G2, overprotective parenting might increase the risk of peer problems in G2; thus, specific types of parenting would function as a transmission belt. In short, multiple‐generation cohorts with data on G1 childhood and adolescence, parenting, and G2 childhood and adolescence assessed at the same age as for G1 are very useful for studying continuity across generations, and mediating and moderating factors that explain, strengthen, or weaken transmission.

## Genetic and Environmental Influences

Although focusing on parenting as a potential transmission pathway helps researchers understand intergenerational continuity, genetic transmission likely also plays a role in the scenarios described earlier: Genetically influenced characteristics might contribute to peer experiences, parenting, and mental health, and might partly explain intergenerational continuity through genetic transmission. Disentangling genetic and environmental transmission pathways has traditionally required twin and children‐of‐twin designs, but genetically informed multiple‐generational cohorts are also suitable. For instance, researchers can test the influence of transmitted (i.e., direct genetic) and nontransmitted genetic material on the development of offspring. That is, G1 and G2 resemble each other genetically to about 50% of DNA, but nontransmitted alleles still influence G2 through environmental pathways. In the Dunedin study, researchers demonstrated this influence of parents’ genetic makeup on the caregiving environment they provided for their offspring (Wertz et al., 2019). These analyses could be extended by exploring how the genes and behavior of offspring influence parenting; this would help researchers understand the influence of genes relative to that of preconception characteristics. Finally, epigenetic research that seeks to understand how experiences in one generation are biologically transmitted to and unfold in the next generation depends on multiple‐generation data.

## Looking Ahead and Practical Implications

In the research questions I have discussed in this article, I have focused on mental health and social development, but the spectrum of topics that can be addressed with multiple‐generation cohort data is broader. For instance, parenting is associated with the socioeconomic status of the family of origin (McAnally et al., 2021) and marital relationship quality which, in turn, are linked to how one experienced one’s parents’ marriage. With respect to intergenerational transmission, stability and mobility in educational attainment across generations is a research topic that has long been of interest to sociologists. Multiple‐generation cohorts allow researchers to focus on transmission pathways in the developmental tradition, such as cognitively stimulating parenting. Educational attainment is also a very relevant outcome for those interested in genetic and environmental contributions to child development.

Moreover, multiple‐generation cohorts can help elucidate the interplay of parents’ body mass index, lifestyle, and genetic material in predicting the risk to offspring of obesity. Other novel research questions that can be tackled with multiple‐generation cohort data include genetic moderation of preconception influences on parenting and shared genetic origins of both. Finally, because multiple‐generation cohorts recruit from a pool of male and female participants and often include the other biological parent, they are well suited to helping researchers learn more about the parenting fathers provide and the determinants of individual differences therein.

If parents’ mental health (Spry et al., 2018) and parent–child bonding (Borschmann et al., 2019) are negatively affected by preconception factors and offspring are at greater risk for psychopathology if their parents experienced early‐onset depression (Jaffee et al., 2020), insights from multiple‐generation cohorts have practical implications. Child and adolescent histories affect future health, well‐being, and relationships, and can exert a domino effect on offspring. Intervening before mental health problems recur or childhood socioeconomic circumstances harm academic attainment and future employment opportunities also benefits future generations. Moreover, family doctors, as well as child and family welfare service practitioners, could assess mental health histories and circumstances of growing up to buffer negative effects on parenting and break cycles of transmission. As such, multiple‐generation cohorts can help researchers as well as practitioners.

## Challenges of Multiple‐Generation Cohorts

Although they are clearly valuable, multiple‐generation cohorts in which G2 are assessed at specified ages rather than brought to the lab during G1 assessments are challenging in terms of logistics and analyses. Births of G2 cannot be planned, and new offspring might enter the study over an extended period. For instance, in the Dunedin study, which assessed G2 at 3 and 15 years, since babies were born to participants younger than 20 years and older than 40 years (Jaffee et al., 2020), G2 entered the study for more than 20 years. This calculation does not even account for male participants who might continue to father new babies for many more years. Not only is it rare for funding bodies to commit to studies that run for decades, but in this extended timeframe, instruments can become outdated.

This continuous accumulation of data also poses analytical challenges. Results might change with better‐powered analyses, and recruiting G2 is not random and may lead to biases, especially as early entries are born to younger parents than late entries. Thus, studying multiple‐generation cohorts requires a tailored strategy to determine when data are analyzed. Whereas researchers might use power analysis to determine the sufficient size of a sample for an expected effect, the multiple‐generation design is innovative, which impedes the determination of an expected effect size. Also, researchers should not simply hope for the best and analyze data without regard to the size and characteristics of the sample collected up to a particular moment.

Studies that use multiple‐generation data are sometimes based on selected groups such as young mothers (Pearson et al., 2018), or include families with G2 born during a particular period that reflects G1 peak age of fertility (Borschmann et al., 2019; Spry, Moreno‐Betancur, et al., 2020) or updated analyses when additional data became available (Belsky, Hancox, Sligo, & Poulton, 2012; Belsky, Jaffee, Sligo, Woodward, & Silva, 2005). Another way to deal with changing sample size is sequential Bayes factor design, in which the Bayes factor quantifies relative support for the null and alternative hypotheses and indicates which of the contrasting hypotheses are more supported by the available data (Hoijtink, Mulder, van Lissa, & Gu, 2019). The Bayesian approach implies that previously known information is incorporated into analyses and knowledge is updated as new information is acquired (van de Schoot et al., 2014). As such, evidence accumulates toward the population value as the Bayes factor is updated again and again. In practice, this means that data can be analyzed repeatedly to understand whether additional data points (e.g., G2 entering the study) affect the Bayes Factor in the direction of the null or alternative hypothesis, and continues until a predetermined Bayes factor threshold has been reached. Naturally, full transparency is inevitable when using this approach and researchers should preregister planned analyses and thresholds. To account for bias introduced by nonrandom entry into the study, researchers could split the sample according to parental age and examine whether results differ.

In addition to the difficulty of planning related to the duration of data collection, multiple‐generation designs pose other challenges. For instance, when one parent is involved with the study, the parent who did not enter the study with the original cohort might not consent to participation, which could affect sample sizes for analyses; it could also raise questions about whether data about a person that is collected from someone else can be used and whether partner selection into the study is biased. However, researchers must attempt to include the other parent and information on his or her developmental histories to comprehensively study risk transmission from G1 to G2, including the role of assortative mating where risk factors in both parents’ histories might be correlated. Even if information on the developmental history of the other parent is available, preconception information will usually be retrospective, which would hamper research on the effects of the interplay of both parents’ developmental histories on parenting and child development.

For several reasons, G2 cohorts are hardly representative: First, the G1 cohort might already be a select sample at the start of a study and attrition over time is not random, leaving a specific group of G1 to potentially enter offspring studies. Second, not all of those in the longitudinal sample will agree to participate in the offspring study. Researchers may find it difficult to ascertain whether nonparticipation is due to not having children or not responding to invitations to participate in the spinoff. Third, demographics in a population can change substantially over time, but a study may include only offspring of a sample that was recruited (and representative of the population) decades ago. In addition to reducing the generalizability of findings, these limitations bias comparisons between G1 and G2 (e.g., with regard to mental health and educational attainment) and might affect associations between preconception factors and outcomes.

Whereas these challenges apply to multiple‐generation cohorts specifically, the studies carry the same limitations as apply to any observational cohort. For instance, it is impossible to disentangle cause and consequence, and confounding factors play a role. Genetically informed cohorts can, to some extent, control for part of the covariance that is the result of shared genetic origins, but other confounders might be unobserved.

Finally, this article also has limitations. I included cohorts in which the focal sample consisted of boys and girls, and collected information up to at least adolescent development to overcome limitations of pregnancy cohorts that include only biological mothers and to allow for comprehensive research into preconception factors. I emphasized cohorts with information on G2 child development and parenting, leaving out studies with a more epidemiological focus. Moreover, although I made great effort to uncover all multiple‐generation studies by gathering information from different sources, some suitable cohorts may have been left out. This likely occurred because data collection is ongoing and findings have not been published, possibly because of the challenges of publishing preliminary results highlighted earlier. Given the relevance of such data for understanding development, researchers should try to publicize information as preliminary cohort profiles or on study websites.

## Conclusion

In this article, I sought to demonstrate the value of multiple‐generation cohort data for developmental researchers and encourage their use in research on parenting and child development. The use of such cohorts, which are most informative when G2 assessments are conducted at specific ages of offspring rather than being considered “by‐catch” during regular parent assessments, is becoming more common as ongoing longitudinal cohorts enter parenthood. This is helpful for researchers studying parenting and child development. Such cohorts can provide information on how child and adolescent experiences affect parenting and genetic and environmental pathways of intergenerational transmission because they allow individual differences in parenting and experiences in parenthood to be traced back along developmental histories and recognize that the origins of child development lay partly in parents’ past. As such, multiple‐generation cohorts have much to offer and contribute uniquely to our toolbox for developmental research.

## Supporting information


**Appendix S1.** Information on cohorts included in Figure 1.

## Footnotes

^1^ I selected these cohorts based on an extensive search of cohort profiles in the *International Journal of Epidemiology*, a Twitter call, and personal communication with colleagues who are experts on such data. This strategy should mean that most multiple‐generation cohorts with data of interest to developmental researchers are included.
