# Supplementary material for: The Value of Multiple‐Generation Cohorts for Studying Parenting and Child Development
Source: Child Dev Perspect. 2021 Mar 22;15(2):83–9. doi: 10.1111/cdep.12403 (PMC8251532; doi:10.1111/cdep.12403)
Supplement: Supplementary file 1 — Appendix S1. Information on cohorts included in Figure 1. [file CDEP-15-83-s001.docx]

**Supplementary Material**

**Information on cohorts included in Figure 1**

**Original cohort: Avon Longitudinal Study of Parents and Children (ALSPAC)**

ALSPAC initially included over 14000 expecting mothers (G0) who were representative of the population of southwest England at the time but more likely to be white and of slightly higher SES than a national comparison sample, especially those who were retained in the sample (Boyd et al., 2013).

**Next generation: ALSPAC-G2**

<http://www.bristol.ac.uk/alspac/>

**Original cohort: Dunedin Multidisciplinary Health and Development Study**

The Dunedin Study initially included over 1000 participants who were representative of the population of New Zealand’s South Island at the time including with respect to SES and percentage of participants identifying as Māori (Poulton, Moffitt, & Silva, 2015).

**Next generation: The Parenting Study (age 3), The Next Generation Study**

<https://dunedinstudy.otago.ac.nz>

**Original cohort: Australian Temperament Project (ATP)**

The Australian Temperament Project initially included almost 2500 participants and is representative for the population of Victoria, Australia at the time the project was initiated. Parents of focal participants were relatively well-educated, worked mostly in skilled and professional occupations and 20% of mothers and 27% of fathers were born in a variety of countries other than Australia, including European, Middle Eastern, and Asian countries.

**Next generation: Australian Temperament Project Generation 3 (ATPG3)**

<https://www.melbournechildrens.com/atp/>

**Original cohort: TRacking Adolescents Individual Lives Survey (TRAILS)**

TRAILS initially included over 2700 participants who came from families with slightly more highly-educated parents and showed higher school performance than non-responders (Oldehinkel et al., 2015). The TRAILS sample includes 11% of participants whose parents were not born in the Netherlands.

**Next generation: TRAILS NEXT**

[www.trails.nl/eng](http://www.trails.nl/eng)

**Original cohort: Victorian Adolescent Health Cohort Study (VAHCS)**

VAHCS initially contained a representative sample of almost 2000 participants, recruited as stratified, random sample in Victoria, Australia. Almost 70% of parents had completed high school or college/university (Rodwell et al., 2018). At the age 35 assessment, the sample was still very similar to a national sample in terms of education and employment (Butterworth, Becker, Degenhardt, Hall, & Patton, 2018)

**Next generation: Victorian Intergenerational Health Cohort Study (VIHCS)**

<https://www.mcri.edu.au/research/projects/2000-stories>

**References Supplementary Material**

Boyd, A., Golding, J., Macleod, J., Lawlor, D. A., Fraser, A., Henderson, J., … Smith, G. D. (2013). Cohort Profile: The ‘Children of the 90s’—The index offspring of the Avon Longitudinal Study of Parents and Children. *International Journal of Epidemiology*, *42*, 111–127. https://doi.org/10.1093/ije/dys064

Butterworth, P., Becker, D., Degenhardt, L., Hall, W. D., & Patton, G. C. (2018). Amphetamine use in the fourth decade of life: Social profiles from a population-based Australian cohort. *Drug and Alcohol Review*, *37*(6), 743–751. doi: 10.1111/dar.12840

Oldehinkel, A. J., Rosmalen, J. G., Buitelaar, J. K., Hoek, H. W., Ormel, J., Raven, D., … others. (2015). Cohort Profile Update: The TRacking Adolescents’ Individual Lives Survey (TRAILS). *International Journal of Epidemiology*, (44(1)), 76–76n. https://doi.org/doi: 10.1093/ije/dyu225

Poulton, R., Moffitt, T. E., & Silva, P. A. (2015). The Dunedin Multidisciplinary Health and Development Study: Overview of the first 40 years, with an eye to the future. *Social Psychiatry and Psychiatric Epidemiology*, *50*(5), 679–693. https://doi.org/10.1007/s00127-015-1048-8

Rodwell, L., Romaniuk, H., Nilsen, W., Carlin, J., Lee, K., & Patton, G. (2018). Adolescent Mental Health and Behavioural Predictors of Being NEET: A Prospective Study of Young Adults Not in Employment, Education, or Training. *Psychological Medicine*, *48*(5), 861–871. doi: 10.1017/S0033291717002434
